# Sll0528, a Site-2-Protease, Is Critically Involved in Cold, Salt and Hyperosmotic Stress Acclimation of Cyanobacterium *Synechocystis* sp. PCC 6803

**DOI:** 10.3390/ijms151222678

**Published:** 2014-12-08

**Authors:** Haijin Lei, Gu Chen, Yuling Wang, Qinglong Ding, Dong Wei

**Affiliations:** College of Light Industry and Food Sciences, South China University of Technology, 381 Wushan Road, Guangzhou 510641, China; E-Mails: lei_hai_jin@163.com (H.L.); wang.yulingwyl@163.com (Y.W.); dingql21@126.com (Q.D.); fewd304@scut.edu.cn (D.W.)

**Keywords:** *Synechocystis* sp. PCC 6803, site-2-proteases, Sll0528, cold stress, salt stress, hyperosmotic stress, RT-qPCR, knock-out mutant, *in vitro* protease activity

## Abstract

Site-2-proteases (S2Ps) mediated proteolysis of transmembrane transcriptional regulators is a conserved mechanism to regulate transmembrane signaling. The universal presence of S2P homologs in different cyanobacterial genomes suggest conserved and fundamental functions, though limited data has been available. Here we provide the first evidence that Sll0528, a site-2-protease in *Synechocystis* sp. PCC 6803 is crucial for salt, cold and hyperosmotic stress acclimation. Remarkable induction of *sll0528* gene expression was observed under salt, cold and hyperosmotic stress, much higher than induction of the other three S2Ps. Knock-out of *sll0528* gene in wild type *Synechocystis* sp. PCC 6803 increased their sensitivity to salt, cold and hyperosmotic stress, as revealed by retarded growth, reduced pigments and disrupted photosystems. The *sll0528* gene was induced to a much smaller extent by high light and mixotrophic growth with glucose. Similar growth responses of the *sll0528* knockout mutant and wild type under high light and mixotrophic growth indicated that *sll0528* was dispensable for these conditions. Recombinant Sll0528 protein could cleave beta-casein into smaller fragments. These results together suggest that the Sll0528 metalloprotease plays a role in the stress response and lays the foundation for further investigation of its mechanism, as well as providing hints for the functional analysis of other S2Ps in cyanobacteria.

## 1. Introduction

As the oldest and most abundant photosynthetic organisms, cyanobacteria (blue-green algae) play essential roles in ecology and biogeochemistry. Their stress responses are good examples for indicating how organisms adapt to an ever-changing environment. Recently, cyanobacteria have attracted considerable attention as a sustainable resource of energy and biomolecules due to their photosynthetic conversion of CO_2_, water and sunlight into bio-based fuels and chemicals [1,2,3]. Therefore, it is important to understand the stress response of cyanobacteria for scientific interests as well as for biotechnological applications.

In cyanobacterium *Synechocystis* sp. PCC 6803 (hereafter *Synechocystis*), two-component regulatory systems, serine/threonine protein kinases, and transcription factors, as well as RNA polymerase sigma factor (σ) subunits are revealed as stress sensors and signal transducers during stress response [4]. Nonetheless, many questions remain. For example, how are sigma factors activated during the stress response? Among the known mechanisms of transmembrane signaling, the Site-2-proteases (S2Ps) mediated proteolysis of transmembrane transcriptional regulators is widespread and conserved among different organisms [5]. Site-2 proteases are named after the founding member of this protease family, human S2P. Human S2P activates cholesterol and fatty acid biosynthesis by cleaving transcription factor SREBP (Sterol Regulatory Element Binding Protein) after site-1 protease (S1P) cleavage [6,7]. S2P homologs are multiple transmembrane proteins with a conserved HExxH active motif within a transmembrane domain and an NPDG motif in another transmembrane domain. S2Ps are widely identified in bacteria, fungi, plant and animals, suggesting their conserved and essential functions in signal transduction [5,8]. We have characterized S2P homologs in photosynthetic organisms, such as EGY1 and EGY2 in *Arabidopsis*, and Slr0643 in *Synechocystis* [9,10,11,12]. EGY1, a chloroplast membrane-bound metalloprotease, is required for thylakoid grana development and accumulation of chlorophyll and chlorophyll a/b binding proteins [9]. EGY1 is also involved in regulation of endodermal plastid size and number, as well as the stimulatory effect of ethylene on hypocotyl gravitropism [13]. EGY2, another chloroplast-located Arabidopsis S2P, plays a role in hypocotyl elongation [10]. Slr0643 is essential for acid acclimation in *Synechocystis* probably through the activation of SigH [12].

At least one, and usually more than one S2P was identified in all of the presently available cyanobacterial genomes, including the reduced genomes from marine picoplanktonic strains such as *Synechococcus* and *Prochlorococcus*. The universal presence of S2P homologs in different cyanobacterial genomes suggest conserved and fundamental functions, though limited data has been available. Recently five putative S2Ps in *Anabaena variabilis* were reported [14], but their physiological functions remain enigmatic, and none of their *in vivo* substrates are revealed. 

Thus, here in this study, we first investigated the gene expression profile of four S2Ps (Sll0528, Slr0643, Sll0862 and Slr1821) of *Synechocystis* under five conditions, namely salt, cold, hyperosmotic stress, high light and mixotrophic growth. Then the function of *sll0528*, the gene which was the most dramatically induced upon stress, was further explored through its knockout mutant and *in vitro* protease activity assay. Results indicated that the metalloprotease Sll0528 plays an essential role in salt, cold and hyperosmotic stress acclimation of *Synechocystis*.

## 2. Results and Discussion

### 2.1. Expression Profile of S2Ps under Different Conditions

**Figure 1:**
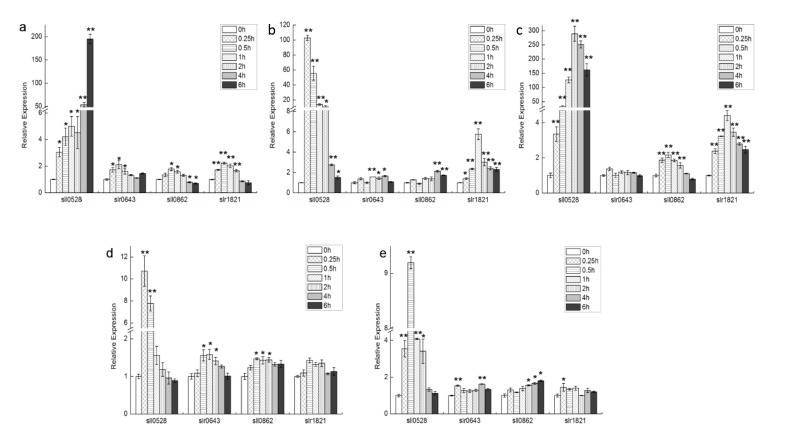
Transcriptional profiles of four S2P homologs under different conditions. Time course expression profile of *sll0528*, *slr0643*, *sll0862* and *slr1821* genes under salt (**a**); hyperosmotic stress (**b**); cold (**c**); high light (**d**) and mixotrophic growth (**e**) were analyzed by quantitative real-time RT-PCR. Significant differences were indicated as * for *p* < 0.05, and ** for *p* < 0.01.

Gene expression profiles of four S2P genes of *Synechocystis*, *sll0528*, *slr0643*, *sll0862* and *slr1821*, were investigated under five conditions, namely salt, hyperosmotic stress, cold, high light and mixotrophic growth (Figure 1).

Under salt stress with 854 mM NaCl, expression level of *sll0528* kept increasing intensively from 0.25 to 6 h, with an expression ratio of more than 50-fold at 4 to 6 h (Figure 1a). The induction patterns of *slr0643*, *sll0862* and *slr1821* genes were similar, with maximal induction at 0.5 h. But their maximal induction was less than 3-fold and much smaller than the induction in *sll0528*. More than forty-fold induction of *sll0528* was previously identified when incubated with 500 mM NaCl for 30 min, 684 mM NaCl for 2 h or 500 mM NaCl for 20 min [15,16,17]. Such consistent and remarkable induction of *sll0528* at different salt concentrations and different experimental duration suggested that *sll0528* was actively involved in salt stress acclimation.

Prompt and sharp *sll0528* induction was observed under hyperosmotic stress generated by 0.5 M sorbitol (Figure 1b). Its expression peaked as 100-fold at 0.25 h and gradually decreased to around the basal level at 6 h. The *slr1821* expression was also enhanced with a maximum 5.5-fold upregulation at 1 h. Hyperosmotic induction of *sll0528* was reported previously at a varied extent, as 4-, 20- or 40-fold induction upon 0.5 M sorbitol in different reports [15,18,19]. Different extents of sorbitol induction might be due to different experimental conditions used by different groups and different time points of observation upon sorbitol treatment. 

Upon transferring to 4 °C, a huge induction was observed in *sll0528* expression, with more than 100-fold upregulation from 1 to 6 h, which peaked at around 2–4 h (Figure 1c). Compared with *sll0528*, *sll0862* and *slr1821* have relatively small but significant upregulation, with maximal induction at 0.5 and 1 h respectively. The relative expression level of *slr0643* was almost unchanged at 4 °C. Compared with the literature, induction of *sll0528* observed here was much higher than the previously reported three times upregulation after transfer from 34 to 22 °C [20]. Fourier transform infrared spectrometry indicated that lipids in plasma membranes were rigidified with decreases in temperature, and cold inducibility of *sll0528* was enhanced upon rigidification of membrane lipids by knocking out the fatty acid desaturases [20]. Thus it was reasoned that higher rigidification of membrane upon lower temperature in our study might partially explain the enhanced *sll0528* inducibility at 4 °C. Further, the sensitivity of *sll0528* induction to temperature decrease might imply its active role in cold stress acclimation.

Upon exposure to high light (200 μmol·photons·m^−2^·s^−1^), an about 10-fold induction was observed in the *sll0528* gene at 0.25 h, but the expression then dropped to basal level at around 2 h (Figure 1d). Compared with the more than 100-fold induction of *sll0528* under cold, salt and hyperosmotic stress, the high light induction of *sll0528* was relatively small and transient. At the same time, only slight induction (less than 1.6-fold) was found in other S2Ps during 0.5 to 2 h, indicating they might not be the primary responsive elements for high light adaption. 

Similar with the situation upon high light, a relative small and transient induction of *sll0528* was found in response to glucose mixotrophism (2.5 mM glucose) (Figure 1e). Its expression peaked at 0.5 h as 9-fold and decreased back to basal level at 4 h. 

### 2.2. Construction of the sll0528 Knockout Mutant

**Figure 2:**
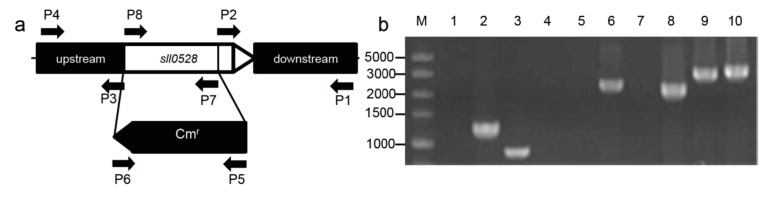
Construction of the *sll0528* mutant. (**a**) The coding region of the *sll0528* gene was replaced with a chloramphenicol resistance gene cassette (Cm^r^) obtained from pACYC184. The arrows P1 to P8 indicated the primers used in PCR verification; (**b**) PCR verification of the *sll0528* mutant. Template was genomic DNA from wild type (lanes 1, 3, 5, 7, 9) or *sll0528* mutant (lanes 2, 4, 6, 8, 10). Primers used as indicated (**a**) were P5 + P6 (lanes 1, 2), P7 + P8 (lanes 3, 4), P1 + P6 (lanes 5, 6), P4 + P5 (lanes 7, 8) and P1 + P4 (lanes 9, 10). PCR products were sequenced to confirm their authenticity.

Due to the strongest induction of *sll0528* gene expression with different stress treatments, it was selected for detailed characterization. A knockout mutant was generated for further physiological characterization under different stress conditions. The *sll0528* gene was disrupted by replacing most of its coding region with a chloramphenicol resistance cassette (Cm^r^) (Figure 2a). After intensive selection under photoautotrophic growth, a completely segregated mutant of *sll0528* was obtained and verified by PCR and sequencing of PCR products (Figure 2b). The corrected insertion of chloramphenicol resistance cassette (Cm^r^) was further confirmed by Thermal asymmetric interlaced PCR (TAIL-PCR). In searching for the flanking region of the inserted Cm^r^ gene, only the upstream and downstream regions of the *sll0528* gene were retrieved in TAIL-PCR using different degenerate primers. RT-PCR in mutants indicated that no *sll0528* transcripts could be detected and confirmed that the *sll0528* gene was successfully disrupted (data not shown).

### 2.3. Varied Phenotype of sll0528 Mutant under Different Conditions

**Figure 3:**
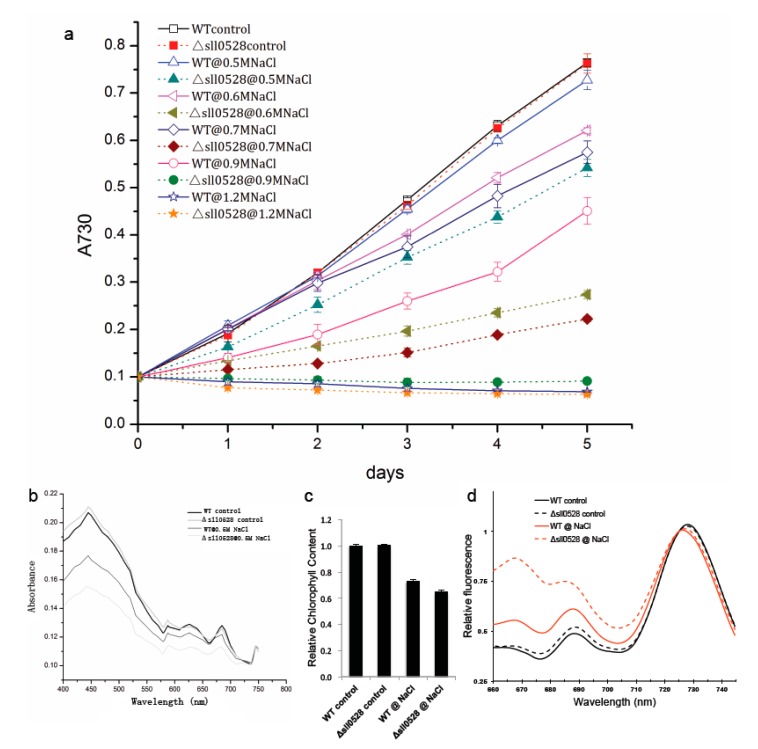
Increased sensitivity of *sll0528* mutant to salt stress. (**a**) Growth curve of wild type (WT) and *sll0528* mutant (Δ*sll0528*) at different concentration of NaCl (0.5, 0.6, 0.7, 0.9, and 1.2 M respectively). Significant differences were observed between mutant and wild type from the 2nd day to 5th day at NaCl of 0.5, 0.6, 0.7 and 0.9 M (*p* < 0.05). Initial OD_730_ was 0.1. Each data point represents the mean of at least three independent biological replicates, and the error bars denote SD; (**b**) Absorption spectra of wild type and *sll0528* mutant 24 h after transfer to 0.5 M NaCl, normalized to OD_730_; (**c**) Relative chlorophyll content of wild type and mutant; (**d**) The 77 K fluorescence emission spectra of wild type and mutant with excitation at 435 nm. Spectra were normalized to emission at 725 nm.

**Figure 4:**
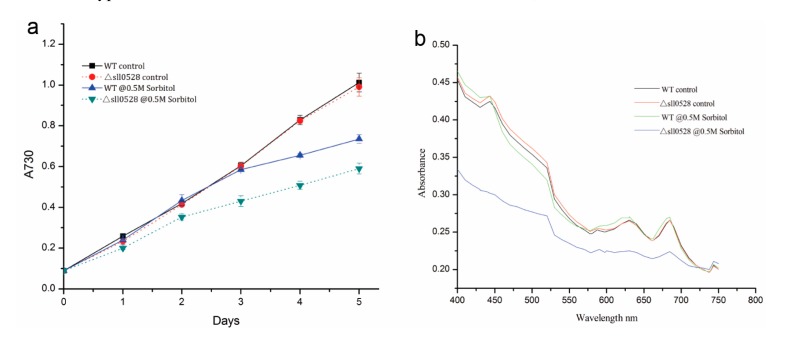
Increased sensitivity of *sll0528* mutant to hyperosmotic stress generated by sorbitol. (**a**) Growth curve of wild type (WT) and *sll0528* mutant (Δ*sll0528*) under 0.5 M sorbitol. Initial OD_730_ was 0.1. Each data point represents the mean of at least three independent biological replicates, and the error bars denote SD; (**b**) Absorption spectra of wild type and *sll0528* mutant 24 h after transferred to 0.5 M sorbitol, normalized to OD_730_.

The *sll0528* mutant grew as well as wild type under optimum culture condition (Figure 3a and Figure 4a), suggesting the Sll0528 protein was dispensable for cell viability at optimized conditions. But the *sll0528* mutant displayed varied responses to different conditions.

#### 2.3.1. Sll0528 Is Crucial for Acclimation to Salt Stress

The acclimation of wild type and the *sll0528* mutant upon salt stress was investigated under 0.5–1.2 M NaCl (Figure 3a). Addition of 0.5 M NaCl in BG11 medium only impeded the growth of wild type insignificantly. But NaCl of 0.6 to 0.9 M notably retarded its growth and 1.2 M NaCl completely blocked the growth of wild type. The growth rate of wild type was nearly halved at 0.9 M NaCl. Compared with wild type, the *sll0528* mutant was much more sensitive to salt stress (Figure 3a). NaCl of 0.5 M impeded the mutant growth significantly, and growth rate under 0.6 M NaCl was less than half of the control. No growth was detected in the mutant even under 0.9 M NaCl.

Furthermore, the whole-cell absorption spectra of wild type and mutant were compared (Figure 3b). The representative absorption peaks for chlorophyll (440 and 680 nm), carotenoids (480–500 nm) and phycocyanin (600–650 nm) were lower in wild type upon salt stress. However, the reduction was much more severe in the *sll0528* mutant. This indicated that accumulation of light-harvesting antenna pigment was severely affected. Further extraction and analysis of chlorophyll content revealed that the reduction of chlorophyll under salt stress was much more severe in mutant than wild type (Figure 3c) and it was consistent with absorption spectra results. Then the fluorescence properties of photosystem I (PSI) and II (PSII) were analyzed by 77 K fluorescence spectroscopy. Excitation of chlorophyll at 435 nm resulted in emission peaks at 685, 695 and 725 nm corresponding to PSII-CP43, PSII-CP47 and PSI, respectively. The PSI/PSII ratio was similar between wild type and mutant under optimal condition. However, the salt induced decrease of the PSI/PSII ratio was much bigger in mutant than in wild type (Figure 3d), suggesting that the photosystem in the *sll0528* mutant was seriously impaired upon salt stress. The seriously impaired photosystems in the *sll0528* mutant might partially explain its retarded, even halted growth under NaCl concentrations that were tolerated by the wild type. 

Marin *et al.* [16] reported that no significant reduction in salt resistance was observed in the completely segregated *sll0528* mutants. The distinction with our observation might due to different salt concentration and culture condition used or different genomic background of the mutant. Re-sequencing of *Synechocystis* had revealed the genomic diversity among different strains used around the world [21,22,23].

Salt stress is one of the environmental factors that limit growth and productivity of organism. As reviewed, so far the mechanisms known for cyanobacteria salt acclimation involve active extrusion of toxic inorganic ions and accumulation of compatible solutes, mainly though *de novo* synthesis [24]. Recently, exopolysaccharides were also suggested in the protection against salt stress [25]. And several stress sensors or signal transducers, such as two-component regulatory systems (Hik/Rre-pair), transcription factors, and RNA polymerase sigma factor (σ) were suggested to contribute to salt acclimation [24,26]. Disruption of the *sll0528* gene remarkably increased the sensitivity of *Synechocystis* to salt stress, which implied that functional Sll0528 protein was crucial for efficient salt acclimation. Research is ongoing in our lab to explore how the functional Sll0528 protein cooperates with the known sensors and signal transducers in salt acclimation.

#### 2.3.2. Sll0528 Is Indispensable for Acclimation to Hyperosmotic Stress

Hyperosmotic stress generated by 0.5 M sorbitol impeded the growth of wild type, while the *sll0528* mutant was more sensitive to 0.5 M sorbitol and grew even more slowly (Figure 4a). Whole-cell absorption spectra of wild type and mutant 24 h under 0.5 M sorbitol indicated the remarkable change in the mutant (Figure 4b). Only slight reduction of carotenoids was found in wild type upon hyperosmotic stress, while dramatic reduction of chlorophyll, carotenoids and phycocyanin were found in the mutant (Figure 4b). The nearly flat absorption spectra of the mutant indicated loss of the characteristic photosynthetic pigment and severely impaired photosystems, which were correlated with its disrupted cell growth (Figure 4). 

There is some overlap in the acclimation to salt and hyperosmotic stress since both of them increase the water potential inside the cell. But hyperosmotic stress does not generate as much ionic stress as salt stress. It was reported that hyperosmotic stress utilized the identical Hik-Rre two component systems as salt stress in perception and signal transduction, but regulated individual genes to different extents [17,18]. Here in our studies, increased sensitivity was found in the *sll0528* mutant to both salt and hyperosmotic stress (Figure 3 and Figure 4). Whether they are due to the same mechanism awaits further investigation. 

#### 2.3.3. Sll0528 Is Crucial for Cold Acclimation

**Figure 5:**
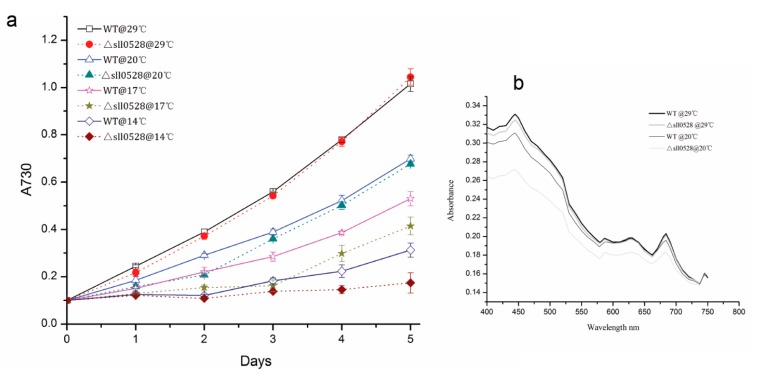
Increased sensitivity of *sll0528* mutant to cold stress. (**a**) Growth curve of wild type (WT) and *sll0528* mutant (Δ*sll0528*) at different temperatures. Significant differences were observed between the mutant and wild type at the 2nd day upon 20 °C, from the 2nd to 5th day upon 17 °C and at the 4th and 5th day upon 14 °C respectively (*p* < 0.05). Initial OD_730_ was 0.1 when the temperature was changed. Each data point represents the mean of at least three independent biological replicates, and the error bars denote SD; (**b**) Absorption spectra of wild type and *sll0528* mutant 24 h after transferred to 20 °C, normalized to OD_730_.

The growth rate of wild type decreased gradually when the temperature decreased from 29 to 14 °C. As compared to wild type, the *sll0528* mutant had a delayed or disrupted cold acclimation (Figure 5a). Under 20 °C, the *sll0528* mutant grew more slowly than wild type during the first two days, but it caught up with wild type from the third day and had a similar growth rate with wild type on the fourth and fifth days. The whole-cell absorption spectra of wild type and mutant 24 h after being transferred to 20 °C indicates the distinct variation in the mutant (Figure 5b). Compared to the slight reduction of chlorophyll and carotenoids in wild type upon cold stress, a remarkable decrease of chlorophyll, carotenoids and phycocyanin in mutant upon cold stress suggested severely impaired photosystems. These defects were correlated with its retarded cell growth. Under lower temperature such as 17 °C, no significant growth was observed in the mutant during the first three days, but it resumed growth from the third day and had similar growth rate as wild type on the fourth and fifth days, though the cell density was still lower than wild type. At 14 °C, no propagation of wild type or mutant was detected in the first two days. Wild type resumed growth slightly on the third day, and significant growth was detected on the fourth and fifth days. However, the mutant could hardly recover during the five days of our observation. Thus, such postponed or disrupted cold acclimation in the *sll0528* mutant implied its reduction in cold resistance, and suggested the functional Sll0528 protein was essential for efficient cold acclimation. 

At lower temperature, *Synechocystis* activated the expression of the fatty acid desaturases gene and enhanced membrane lipid unsaturation to ensure membrane fluidity [27,28,29]. Further, histidine kinase Hik33 was an important sensor of cold stress, which might sense the membrane fluidity [30,31]. RNA-binding protein 1 (Rbp1) and RNA helicase (CrhR) were recently reported to play indispensable roles in cold acclimation [32,33,34,35,36]. Whether the membrane fluidity and the extent of membrane lipid unsaturation of *sll0528* mutant were different from wild type upon cold stress needs to be further investigated. Also, exploration of the relationships between Sll0528 and Hik33, as well as Rbp1 and CrhR might help to sketch the picture of cold acclimation in *Synechocystis*. 

#### 2.3.4. Sll0528 Is Dispensable for Acclimation to High Light and Mixotrophic Growth

**Figure 6:**
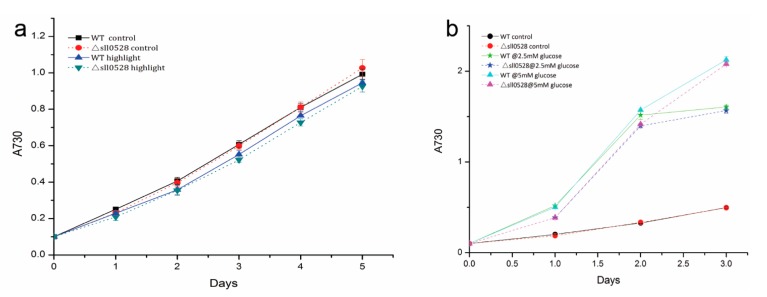
(**a**) Growth curve of wild type (WT) and *sll0528* mutant (Δ*sll0528*) in continuous light of control (30 μmol·m^−2^·s^−1^) and high light (150 μmol·m^−2^·s^−1^); (**b**) Growth curves of wild type and *sll0528* mutant under mixotrophic growth condition with different concentration of glucose. Initial OD_730_ was 0.1 when the condition was changed. Each data point represents the mean of at least three independent biological replicates, and the error bars denote SD.

The *sll0528* mutant had a growth rate similar with wild type under continuous illumination of 30 μmol·m^−2^·s^−1^. The growth rate of both wild type and *sll0528* mutant was slightly decreased under higher light illumination of 150 μmol·m^−2^·s^−1^. But the decrease was not significant and there is no notable difference between wild type and the *sll0528* mutant (Figure 6a). 

Under mixotrophic growth condition, glucose was found to accelerate growth of wild type and the *sll0528* mutant remarkably (Figure 6b). As to wild type, glucose of 2.5 or 5 mM doubled the OD_730_ at 24 h, and tripled its OD_730_ at 48 h. After 48 h, the growth rate under 2.5 mM glucose slowed down and was similar with control under autotrophic growth, while the growth rate under 5 mM glucose still surpassed autotrophic control. This might be due to the exhaustion of glucose under 2.5 mM, but not 5 mM glucose. As for the *sll0528* mutant, it had a similar accelerated growth pattern as wild type under mixotrophic growth condition. Though its growth rate was slightly lower than wild type under 2.5 and 5 mM glucose, the difference was not significant (Figure 6b).

The similarity of response to high light and mixotrophic growth condition in the *sll0528* mutant and wild type suggested that although the *sll0528* gene was induced (Figure 1d,e), the Sll0528 protein was dispensable for these conditions.

### 2.4. Recombinant Sll0528 Has Proteolytic Activity

**Figure 7:**
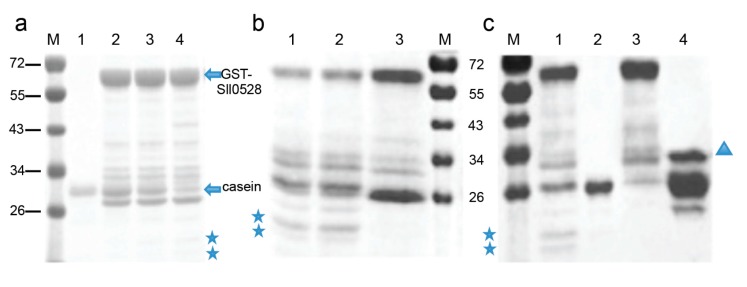
*In vitro* protease activity of recombinant Sll0528. (**a**) The time-dependent proteolytic activity of recombinant Sll0528. Lane 1, the substrate beta-casein remained intact in the reaction buffer after 15 h of incubation. Lane 2, reaction mixture of GST-Sll0528 and beta-casein prior to incubation. Lanes 3 and 4, incubated with GST-Sll0528 for 5 and 15 h, the substrate beta-casein was cleaved significantly. Stars indicated the cleaved fragments of beta-casein; (**b**) The inhibitor study. Lane 1, GST-Sll0528 cleaved beta-casein significantly after 15 h incubation. Lane 2, the proteolytic activity of Sll0528 was not inhibited by Complete EDTA-free (a cocktail of serine and cysteine protease inhibitors). Lane 3, the proteolytic activity of Sll0528 was inhibited by 10 mM *o*-phenanthroline; (**c**) The comparison of proteolytic activity to the recombinant protein P. Lane 1, GST-Sll0528 cleaved beta-casein significantly after 15 h incubation. Lane 2, the substrate beta-casein remained intact in the reaction buffer after 15 h of incubation. Lane 3, purified GST-Sll0528 in the reaction buffer after 15 h incubation. Lane 4, reaction mixture of beta-casein and GST-recombinant protein P (indicated by triangle).

To further investigate the proteolytic activity of Sll0528, a GST-Sll0528 construct was generated and expressed in *E. coli*. The recombinant GST-Sll0528 protein was purified and tested against beta-casein. Beta-casein was cleaved into smaller fragments time-dependently when incubated with GST-Sll0528 (Figure 7). Beta-casein could not be digested by another GST recombinant protein P. It indicated that the protease activity was from GST-Sll0528, but not a contaminant from the expression system. This proteolytic activity was not inhibited by Complete EDTA-free (a cocktail of serine and cysteine protease inhibitors), but was inhibited by *o*-phenanthroline (a metal chelator), suggesting that Sll0528 might function as a metalloprotease in *Synechocystis*.

## 3. Experimental Section

### 3.1. Strains, Culture and Stress Conditions

Derived from ATCC27184, *Synechocystis* sp. PCC 6803 was cultivated in BG11 medium and illuminated with 30 μmol·photons·m^−2^·s^−1^ at 30 °C as optimum condition. Cultures were buffered with 20 mM HEPES-NaOH (pH 7.5), with oscillation at 130 rpm. The stress conditions used in RT-qPCR were as follows: salt stress (5% NaCl, 854 mM); cold stress (4 °C with light illumination); hyperosmotic stress (0.5 M sorbitol); high light (200 μmol·photons·m^−2^·s^−1^); mixotrophic growth condition (2.5 mM glucose). Samples of 30 mL before the stress (0 h) and after timely intervals of 0.25, 0.5, 1, 2, 4 and 6 h were collected. Harvested cells were washed once in 1.6 mL of TE (pH 8.0) and centrifuged at 12,000 rpm at 4 °C for 5 min. The cell pellets were immediately frozen in liquid nitrogen and stored at −80 °C until they were used for RNA isolation.

### 3.2. RNA Isolation and Quantitative RT-PCR

Total RNA was extracted according to the instructions of the RNA extraction kit (DongSheng Biotech, Guangzhou, China) with modification, as previously described [12]. The DNA residue was removed by DNase and DNA contamination was tested by PCR reaction without reverse transcription. Quantitative RT-PCR was performed using one-step SYBR Green I kit (TAKARA Biotech, Dalian, China) on ABI7500 (Life Technologies, Grand Island, NY, USA) using the following conditions: reverse transcription program as 42 °C for 5 min, 95 °C for 10 s; amplification and quantification program repeated for 40 cycles of 95 °C for 5 s, 60 °C for 34 s; melting curve program as 95 °C for 15 s, 60 °C for 1 min and 95 °C for 15 s. The *rnpB* gene that encodes subunit B of ribonuclease P was used as an internal control. The primers sets of S2Ps were listed in Table 1. Melting curve analysis was performed after each run to confirm the homogeneity in the amplification. Three independent biological replicates for each sample and three technical replicates of each biological replicate were analyzed. For the reactions, a master mix of the following components was prepared: 10 μL 2× One step SYBR RT-PCR Buffer, 3.2 μL RNase-Free H_2_O, 0.8 μL (0.4 μM) forward primer, 0.8 μL (0.4 μM) reverse primer, 0.8 μL PrimeScript one-step enzyme mix, 0.4 μL ROX, 4 μL total RNA. The relative expression of genes was calculated using 2^−∆∆*C*t^ method [37].

### 3.3. Construction of sll0528 Mutant

The downstream and upstream fragments of *sll0528* gene were amplified from wild-type *Synechocystis* genomic DNA with primer pairs as indicated in Figure 2 and Table 1, P1 and P2, P3 and P4 respectively. The PCR fragments digested with XhoI and SacI or BamHI and NdeI (sites underlined in primer sequences) were inserted into the XhoI-SacI or BamHI-NdeI site of pET-30b (+) to generate p0528. The chloramphenicol resistance cassette was amplified from pACYC184 with primer P5 and P6. It was digested with SacI and BamHI, and inserted into the Sac1-BamH1 site of p0528 to yield pC0528, which was used to transform *Synechocystis*. Chloramphenicol of 40 μg·mL^−1^ was added for the selection of *sll0528* mutant. The complete replacement of *sll0528* coding region was verified with primers P7 and P8. 

### 3.4. Measurement of Physiological Parameters

The growth curve of wild type and mutant was prepared by measuring OD_730_ every 24 h during the first three or five days. Whole cell absorbance was measured 24 h after stress condition (unless otherwise specified) by using a UV2300 spectrophotometer (Techcomp, Beijing, China) scanning from 400 to 750 nm. Extraction and measurement of chlorophyll were performed as described previously [38]. Chlorophyll florescence at 77 K was measured as described previously [39] with an LS-55 fluorescence spectrometer (PerkinElmer, Waltham, MA, USA).

### 3.5. Expression of Recombinant Sll0528 and in Vitro Proteolytic Activity Assay

The coding sequence of Sll0528 was amplified using primers P9 and P10. The fragment was cloned into pGEX-2T to generate a GST-Sll0528 fusion protein. This construct was expressed in *E. coli* strain BL21(DE3)pLysS and recombinant GST-Sll0528 was purified through GST affinity column (Sigma-Aldrich, St. Louis, MO, USA). The proteolytic activity assay was conducted against 1 or 2 μg beta-casein in a reaction buffer containing 50 mM Tris-acetate (pH 8.0), 80 mM NaCl, 5 mM Mg-acetate and 12.5 µM Zn-acetate. Quantification of casein was performed by ImageJ on the Coomassie brilliant blue R250 stained gel. 

### 3.6. Data Analysis

Relative expression levels were determined after normalization with the *rnpB* transcript level using ABI7500 SDS software version 4.0 (Life Technologies, Grand Island, NY, USA). The average relative expression levels and standard deviations were determined from the data generated in independent experiments. One way ANOVA was performed by SPSS software to determine the significance of the relative expression ratios, *p* < 0.05 was considered significant. 

**Table 1:** Primers used in this research. The restriction enzyme cleavage sites were underlined.

Primer Name	Sequence of Primer (5'–3')
sll0528-L	GGAAGCCTTTACTGCTGAAGAT
sll0528-R	TGTCGGCACCAATAACCAAG
sll0862-L	GGCAAATGCGGGAAGAAG
sll0862-R	TGTCACCGAGCACAGTGGT
slr0643-L	GGTTTGTCCACTGCTCTACT
slr0643-R	GGCTGTGATGATTTCTGC
slr1821-L	TTGGATGGTGGGCAATTG
slr1821-R	ATACCCCTAAGCTCAGCAGAAG
rnpB-L	CCACTGAAAAGGGTAAGGG
rnpB-R	CTCCGACCTTGCTTCCA
P1	CGAGCTCCTACGGAAGACATCAAACACG
P2	CCGCTCGAGGGCGAAATGTTGACCTTGAC
P3	GGAATTCCATATGCTGGTTATGGTGGTTTACTGA
P4	CGCGGATCCATGTTAAGAATTGCCTGAGTG
P5	CGCGGATCCTCATCAGTGCCAACATAG
P6	CGAGCTCGGTAAACCAGCAATAGACAT
P7	CAAGTACAC CTAACAGTTGA
P8	ATGTTAAGCCTCAGTTTAG
P9	CGCGGATCCATGTTAAGCCTCAGTTTAGGGG
P10	CCGGAATTCCTAGGCGGCGGAGGTTTGCAG

## 4. Conclusions

In conclusion, the *sll0528* gene was highly induced by salt, cold and hyperosmotic stress and its knockout mutant displayed increased sensitivity to salt, cold and hyperosmotic stress. The recombinant Sll0528 protein was a metalloprotease as its homologs in other organisms. Results here provided the first evidence that Sll0528, a Site-2-protease in *Synechocystis* sp. PCC 6803 is crucial for salt, cold and hyperosmotic stress acclimation. Further investigation of its mechanism will hopefully shed light on the mechanisms of stress acclimation in cyanobacteria.
